# Erratum to: Bacterial pneumonia coinfection and antimicrobial therapy duration in SARS-CoV-2 (COVID-19) infection

**DOI:** 10.1093/jacamr/dlaa095

**Published:** 2020-12-08

**Authors:** Liam Townsend, Gerry Hughes, Colm Kerr, Mary Kelly, Roisin O’Connor, Eileen Sweeney, Catriona Doyle, Ruth O’Riordan, Ignacio Martin-Loeches, Colm Bergin, Ciaran Bannan

*JAC Antimicrob Resist* 2020; **2**: dlaa071

In the original published version of this article, the name of one author, Ignacio Martin-Loeches, was omitted from the author line of this article. This error has now been corrected in the online version of the article.

The authors apologize for this error.

